# Targeting Muscle-Specific E3 Ligases in Cancer Cachexia Through Structure-Based Virtual Screening of Small-Molecule Inhibitors

**DOI:** 10.17912/micropub.biology.002256

**Published:** 2026-10-01

**Authors:** Irfana Muqbil, Jordan D Johnson

**Affiliations:** 1 Department of Natural Sciences, Lawrence Technological University, Southfield, MI USA; 2 Department of Biomedical Engineering, Lawrence Technological University, Southfield, MI USA

## Abstract

Cachexia is a multifactorial metabolic syndrome characterized by severe skeletal muscle loss that cannot be reversed by nutritional support. Common in cancer and other chronic diseases, it significantly increases morbidity and mortality. Muscle wasting is primarily driven by activation of the ubiquitin–proteasome pathway through the E3 ubiquitin ligases MuRF1 and Atrogin-1, which promote protein degradation and muscle atrophy. This study used computational modeling and docking to identify small-molecule inhibitors targeting these ligases, including repurposed drugs with anti-cachectic potential. Thalidomide and EM12-So2F were prioritized based on integrated docking, binding affinity, and drug-likeness/ADME analyses for validation in C2C12 myotube-based cachexia models.

**Figure 1. Computational screening and prioritization of EM12-So2F and thalidomide as candidate ligands of the muscle-specific E3 ubiquitin ligases Atrogin-1 and MuRF1:** (A) DiffDock analysis showing the distribution of smina affinity scores across predicted binding poses for compounds screened against Atrogin-1 (FBXO32) and MuRF1 (TRIM63). EM12-So2F and thalidomide are highlighted as compounds prioritized for further analysis based on integrated consideration of docking pose/confidence, predicted affinity, protein–ligand interactions, and predicted drug-likeness/ADME properties; highlighting does not indicate selection based solely on the most favorable affinity score. (B–C) SwissADME drug-likeness radar plots for EM12-So2F (B) and thalidomide (C). (D–E) Predicted binding poses of EM12-So2F with MuRF1 and Atrogin-1, respectively. (F–G) Predicted binding poses of thalidomide with MuRF1 and Atrogin-1, respectively. Detailed two-dimensional maps of predicted protein–ligand interactions are provided in Extended Data Figure 1. **Table 1.** Smina binding affinity scores (kcal/mol) associated with the highest-confidence DiffDock poses of screened compounds against Atrogin-1 (FBXO32) and MuRF1 (TRIM63). More negative values indicate more favorable predicted binding affinity.

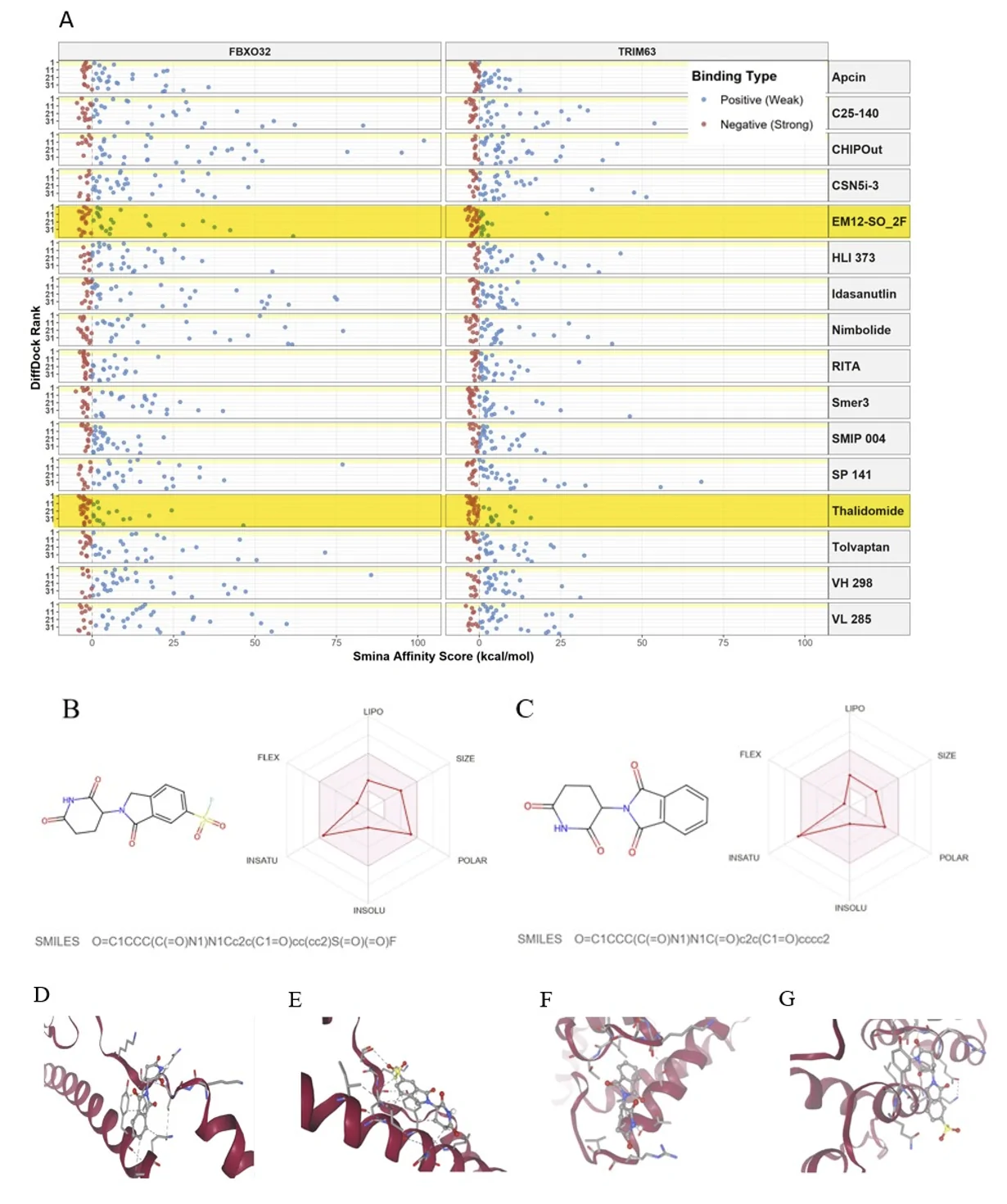



**Table:** 

Drug	FBXO32	TRIM63
Apcin	-1.8641	0.0071
Thalidomide	-4.067	-3.8581
SMER3	-2.985	-1.0228
Nimbolide	12.0342	-1.8091
Tolvaptan	-2.7682	-1.8666
Idasanutlin	-1.887	-1.9223
VL285	16.2867	1.19248
SP141	13.9762	0.06285
RITA	-2.9676	-1.2039
CHIPOut	17.0816	8.8096
SMIP 004	-3.0604	-2.06204
VH298	-2.9676	2.4855
EM12-So2F	-2.4676	-2.8753
C25-140	-3.18	1.161
CSN5o-3	-0.9217	-2.5976
HLI373	2.123	-1.769

## Description

Cachexia or muscle wasting is associated with poor quality of life and is one of the leading causes of morbidity in patients with advanced cancer. Therefore, the identification of novel therapeutic approaches to target cachexia is of critical importance in the management of advanced malignancies. Cancer cachexia is a multifactorial metabolic syndrome characterized by progressive skeletal muscle wasting, with or without adipose tissue loss, that cannot be fully reversed by nutritional supplementation alone (Fearon et al., 2011). It is frequently observed in patients with pancreatic, lung, gastrointestinal, and other advanced malignancies, where it contributes significantly to weakness, fatigue, reduced mobility, poor tolerance to anticancer therapies, and decreased overall survival (Argilés et al., 2014). Despite its major clinical impact, effective therapies for cancer-associated cachexia remain limited.

One of the major molecular mechanisms underlying cachexia is the activation of the ubiquitin–proteasome system (UPS), the primary intracellular pathway responsible for regulated protein degradation. In skeletal muscle, the UPS contributes to accelerated breakdown of structural and contractile proteins during catabolic conditions (Bodine et al., 2001; Gomes et al., 2001). Protein degradation through the UPS involves sequential ubiquitin activation by E1 enzymes, ubiquitin conjugation by E2 enzymes, and substrate-specific ubiquitination mediated by E3 ubiquitin ligases, ultimately directing proteins toward degradation by the 26S proteasome. Among these components, E3 ubiquitin ligases provide substrate specificity and therefore represent attractive therapeutic targets. Two muscle-specific E3 ubiquitin ligases, Muscle RING Finger-1 (MuRF1/TRIM63) and Atrogin-1/FBXO32, are consistently upregulated in experimental and clinical models of cachexia and are considered central mediators of skeletal muscle atrophy (Bodine et al., 2001; Gomes et al., 2001). MuRF1 primarily targets sarcomeric and contractile proteins including myosin heavy chain, whereas Atrogin-1 regulates proteins involved in muscle growth, differentiation, and protein synthesis. Persistent activation of these ligases results in excessive proteolysis, impaired muscle regeneration, and progressive muscle wasting.

Current therapeutic approaches for cachexia mainly focus on nutritional supplementation, exercise, appetite stimulants, and anti-inflammatory interventions; however, these strategies often provide only modest clinical benefit (Baracos et al., 2018). Consequently, there is growing interest in directly targeting the molecular pathways responsible for muscle protein degradation. Inhibition of muscle-specific E3 ligases represents a promising strategy to preserve skeletal muscle integrity and improve outcomes in cachectic patients.

Recent advances in computational biology, molecular modeling, and artificial intelligence-based drug discovery have accelerated the identification of candidate therapeutics against disease-associated targets (Jumper et al., 2021). Computational approaches enable rapid screening of compounds, prediction of protein–ligand interactions, and evaluation of drug-like properties prior to experimental validation. In the present study, we performed in silico screening of multiple E3 ubiquitin ligase inhibitors, including compounds currently in clinical use as well as investigational agents undergoing preclinical evaluation. Protein sequence and structural information were obtained through UniProt, which provides curated protein annotations, domain information, and structural datasets relevant for molecular modeling studies (UniProt Consortium, 2023). Ligand preparation and structural optimization were carried out using Open Babel, enabling conversion and refinement of molecular structures for downstream docking analyses (O’Boyle et al., 2011).

Docking studies were performed using DiffDock, an artificial intelligence–based platform that predicts protein–ligand binding poses and interaction confidence through diffusion generative modeling approaches (Corso et al., 2023). DiffDock enables flexible docking and provides binding confidence estimates that facilitate prioritization of candidate compounds. Additional analyses were conducted using SwissDock to evaluate docking conformations, binding interactions, drug likeness, and pharmacokinetic properties including absorption, distribution, metabolism, and excretion (ADME) characteristics (Grosdidier et al., 2011). Together, these integrated computational approaches provided a robust framework for identifying candidate inhibitors targeting muscle-associated E3 ligases ( Figure 1A –C).

Among the compounds screened thus far, EM12-So2F and thalidomide were prioritized for further investigation based on an integrated assessment of multiple computational parameters rather than predicted binding affinity alone ( Figure 1A –G). Initial DiffDock analysis evaluated predicted binding poses, confidence rankings, and smina affinity scores for the screened compounds ( Figure 1A ; Table 1). Thalidomide showed the most favorable smina affinity scores for both Atrogin-1 and MuRF1, whereas EM12-So2F showed favorable predicted binding, particularly toward MuRF1, but was not the highest-affinity compound for Atrogin-1. Therefore, its prioritization was based on the combined assessment of docking characteristics and subsequent drug-likeness and ADME analyses. SwissADME profiling showed favorable physicochemical and pharmacokinetic characteristics for EM12-So2F and thalidomide ( Figure 1B –C), further supporting their selection. Examination of the predicted binding poses showed that both compounds could be accommodated within predicted binding regions of MuRF1 and Atrogin-1 ( Figure 1D –G), with interaction analysis identifying hydrogen bonds, van der Waals contacts, and hydrophobic interactions with residues surrounding the ligands. Together, these complementary analyses supported prioritization of EM12-So2F and thalidomide for subsequent experimental evaluation. Interestingly, both compounds are established cereblon (CRBN) binders; however, the present study does not establish a mechanistic role for CRBN or PROTAC-mediated activity in their predicted interactions with MuRF1 or Atrogin-1.

Together, these findings demonstrate the utility of integrating structure-based docking, binding affinity prediction, interaction analysis, and drug-likeness/ADME profiling to prioritize candidate compounds targeting muscle-specific E3 ubiquitin ligases. EM12-So2F and thalidomide represent promising candidates identified from the compounds screened thus far, and additional small molecules will be screened using the same computational workflow to expand the pool of potential candidates. Lead compounds emerging from this analysis will undergo target-validation studies to confirm their interaction with and specificity toward MuRF1 and Atrogin-1 before advancing to functional evaluation. Selected lead compounds will then be tested in C2C12 myotube-based models of muscle atrophy to determine whether target modulation translates into preservation of the muscle phenotype. These studies will provide experimental validation of the computational findings and establish whether direct targeting of MuRF1 and Atrogin-1 represents a viable therapeutic strategy for cancer-associated muscle wasting.

## Methods

**Protein and Ligand Preparation**

Protein sequences and structural information for the MuRF1 (TRIM63) coiled-coil domain were obtained from the Protein Data Bank (PDB ID: 4M3L) (Berman et al., 2000; Franke et al., 2014), while sequence and structural information for Atrogin-1 (FBXO32; UniProt accession Q969P5) were retrieved from UniProt, and corresponding structural models were utilized for molecular docking analyses. Candidate E3 ligase inhibitors were selected based on published literature and structural relevance to ubiquitin–proteasome pathway modulation. Ligand structures were retrieved in SMILES or SDF format from chemical databases and converted into optimized three-dimensional conformations using Open Babel for molecular modeling and docking analyses. Protein structures were prepared by removing water molecules and non-essential ligands, followed by optimization of structural geometry prior to docking simulations.

**DiffDock Molecular Docking Analysis**

Protein–ligand docking studies were performed using DiffDock, a generative artificial intelligence–based docking platform that utilizes diffusion modeling to predict ligand-binding poses and protein–ligand interactions. Similar to previously described DiffDock workflows, multiple ligand conformations and docking poses were generated to evaluate translational, rotational, and torsional flexibility during docking inference. Docking outputs included predicted binding poses, confidence score rankings, and smina affinity scores, allowing assessment of both the confidence of the predicted ligand pose and its predicted binding affinity. More negative smina affinity scores were interpreted as more favorable predicted binding affinities. Figure 1A shows the distribution of smina affinity scores across the generated docking poses, whereas Table 1 summarizes the smina affinity score associated with the highest-confidence DiffDock pose for each compound against Atrogin-1 and MuRF1.

**SwissDock Validation and ADME Profiling**

Additional docking analyses and interaction visualization were carried out using SwissDock to further evaluate ligand-binding conformations and intermolecular interactions within predicted binding pockets. Predicted protein–ligand interactions, including hydrogen bonds, van der Waals contacts, and hydrophobic interactions, were examined to characterize the binding environment of prioritized compounds. Drug-likeness, molecular properties, and absorption, distribution, metabolism, and excretion (ADME) characteristics were further assessed to prioritize compounds with favorable pharmacokinetic profiles and therapeutic potential.

**Candidate Prioritization**

Candidate prioritization was based on an integrated assessment of DiffDock confidence ranking, smina-predicted binding affinity, predicted binding poses and protein–ligand interactions, and drug-likeness/ADME properties rather than binding affinity alone. Among the compounds screened thus far, thalidomide and EM12-So2F were prioritized for further investigation based on their combined computational profiles. Thalidomide exhibited the most favorable smina affinity scores toward both Atrogin-1 and MuRF1, whereas EM12-So2F was retained based on the integrated assessment of its docking and predicted pharmacological characteristics despite not exhibiting the most favorable affinity score for Atrogin-1.

## Data Availability

Description: Extended Data Figure 1. Two-dimensional interaction maps of prioritized compounds in the predicted binding regions of MuRF1 and Atrogin-1. (A–B) Predicted interactions of EM12-So2F with MuRF1 and Atrogin-1, respectively. (C–D) Predicted interactions of thalidomide with MuRF1 and Atrogin-1, respectively. The maps show interacting residues and interaction types: conventional hydrogen bonds (green dashed lines), van der Waals contacts (light green), π–sigma interactions (purple), and π–alkyl interactions (pink). These maps provide structural context for the predicted binding poses in Figure 1D–G.. Resource Type: Image. DOI: https://doi.org/10.22002/v1njh-67936
